# Editorial: Integrative and Translational Uses of Herbarium Collections Across Time, Space, and Species

**DOI:** 10.3389/fpls.2020.01319

**Published:** 2020-08-21

**Authors:** Nina Rønsted, Olwen M. Grace, Mark A. Carine

**Affiliations:** ^1^Science and Conservation, National Tropical Botanical Garden, Kalaheo, HI, United States; ^2^Natural History Museum of Denmark, University of Copenhagen, Copenhagen, Denmark; ^3^Comparative Plant & Fungal Biology, Royal Botanic Gardens, Kew, Surrey, United Kingdom; ^4^Department of Life Sciences, The Natural History Museum, London, United Kingdom

**Keywords:** herbarium collections, historical trends, museomics, plant tree of life, uses of herbaria

This Research Topic is dedicated to the legacy of Vicki Funk [1947-2019], who was a Senior Research Botanist and Curator at the Smithsonian’s National Museum for 38 years, and a strong advocate for collections-based research. In 2004, Vicki compiled an inspirational list of uses of herbaria entitled *‘100 Uses for an Herbarium (Well at Least 72)’* (Funk, 2004), a study motivated by her appreciation of the potential for herbaria to make a unique contribution to a remarkable and growing array of research questions, at a time when the long-term survival of many such collections was under threat. The intervening decade and a half has seen that list of uses increase with new and unexpected techniques for the analysis of herbarium specimens and of the data derived from them.

Herbarium collections provide a unique record of our global biodiversity and natural history amassed over centuries. According to Index Herbariorum (Thiers, 2020), a global database of herbaria, as of December 2019, there are 3324 active herbaria in the world that collectively are estimated to hold nearly 400 million specimens. They offer a verifiable source of specimens for a plethora of research questions from taxonomy to evolution and change through time. They are being used to help tackle global societal challenges, and their value is currently increasingly realized and explored (Funk, 2002; Funk, 2004; Bebber et al., 2010; Funk, 2018; James et al., 2018; Meineke et al., 2018). Such impact will deepen as herbarium collections become more accessible both in digital and physical forms, and stories of the specimens and collectors are brought to light. Indeed, current global efforts to digitize herbarium collections are continuously increasing the number of available images and data through portals such as JSTOR Global Plants (www.plants.jstor.org), Global Biodiversity Information Facility (www.GBIF.org), and iDigBio (Integrated Digitized Biocollections; www.idigbio.org) (Soltis, 2017).

As scientists based at three of the world’s oldest and largest herbaria: the Royal Botanic Gardens Kew (K; founded 1852; 8,125,000 specimens), The Natural History Museum, London (BM; founded 1753; 5,200,000 specimens), The Natural History Museum of Denmark (C; founded 1759; 2,900,000 specimens), and one smaller and younger herbarium at the National Tropical Botanical Garden, USA (PTBG; founded 1971; 88,870 specimens), we encourage the continued use and exploration of the unique heritage and resources held in the world’s herbaria.

This Research Topic aims to synthesize and inspire the frontier of integrative and translational research using herbarium collections to highlight their unharvested potential for addressing outstanding research questions and societal challenges. The articles published in this Research Topic provide a selection of examples and new approaches illustrating trends and opportunities in this expanding field.

Though an inherently biased sample of biodiversity through time — shaped by historical and contemporary collecting practices and by colonialism and trade — herbarium specimens provide a verifiable record that a species occurred at a particular place at a particular time, and they represent several hundred years of collection history across the globe (López and Sassone; Romeiras et al.). Their imperfections extend to problems with erroneous identifications and with biased digitization efforts (e.g. Smith and Blagoderov, 2012), but careful assessment of data quality significantly improves their value (Goodwin et al., 2015; Maldonado et al., 2015), as do efforts to evaluate collection history and association with additional data sources (Allasi Canales et al., 2020; López and Sassone; Romeiras et al.; Stefanaki et al., 2019).

These challenges notwithstanding, herbaria provide a unique record of changes in distributions, of extinction and of habitats that have now disappeared. Specimens in herbaria provide readily available information and research possibilities extending far beyond the use of contemporary samples (Greve et al., 2016; Silva et al., 2017; Cardoso et al., 2018) and, indeed, far beyond the scope of what their original collectors may ever have envisaged (Heberling and Isaac, 2017; Heberling et al., 2019).

Herbaria present a time window into the past allowing exploration of changes in composition of floras (Calinger, 2015), of distribution of invasive weeds or threatened species (Stadler et al., 2002; Rivers et al., 2011; Hardion et al., 2014; Martin et al., 2016), changes in flowering times (Davis et al., 2015; Willis et al., 2017), leaf-out times (Everill et al., 2014) or of stomatal densities through time (Large et al., 2017) in response to environmental change, and they can be used to model predictions of future trends (James et al., 2018). Increasingly, they are being used to investigate not just the plant itself but other, associated organisms, in studies of trophic interactions, to infer relationships with associated insects including pollinators and herbivores and with microorganisms including those causing diseases (Martin et al., 2013; Yoshida et al., 2014; Meineke and Davies, 2018; Vega et al.).

Destructive sampling of herbarium samples must always be done with due care and for good scientific reason, but small amounts of material can now be used for a diversity of studies including exploring the viability of seeds from old herbarium specimens for conservation purposes (Godefroid et al., 2011; Porteous et al.; Wolkis and Deans, 2019). As the technical difficulties of extracting high quality DNA from historical materials are being overcome (Wales et al., 2014; Bieker and Martin, 2018) and new high-throughput methods are being developed including a customized Angiosperms353 probe set (Brewer et al.; Johnson et al., 2019), herbarium samples are increasingly being used in genomic studies—often termed Museomics—at all scales, from populations to phylogenies (Kuzmina et al., 2017; Bieker and Martin, 2018; Malakasi et al.) as well as to studies of genome duplications (Viruel et al.), domestication history (Kistler et al., 2018; Ramos-Madrigal et al., 2019), and plant pathogens (Yoshida et al., 2014). Herbarium materials can be a resource of chemical data for chemotaxonomy (Cook et al., 2009; Jafari Foutami et al., 2018; Allasi Canales et al., 2020), chemical ecology (Zangerl and Berenbaum, 2005), environmental bio-indicators (Foan et al., 2010; Monforte et al., 2015; Martinez-Swatson et al.) as well as drug discovery and authentication (Saslis-Lagoudakis et al., 2015; Rønsted et al., 2017).

## Concluding Remarks

Herbaria are a unique resource for understanding global biodiversity and for addressing societal challenges. Their traditional use for taxonomy—for documenting and describing biodiversity – is as important today as it has ever been but that function is being supplemented by an increasing array of questions that herbaria are being used to address. We hope the articles in this Research Topic will inspire new integrative and translational uses of herbarium collections as well as highlight the need for continuous preservation, curation, and expansion of the collections, accompanied by detailed collecting data.

## Author Contributions

NR drafted the editorial with contributions from OG and MC. The authors all contributed to the Research Topic assembly and editing.

## Conflict of Interest

The authors declare that the research was conducted in the absence of any commercial or financial relationships that could be construed as a potential conflict of interest.
